# Supplementary material for: Cryo-EM structure of catalytic ribonucleoprotein complex RNase MRP
Source: Nat Commun. 2020 Jul 10;11:3474. doi: 10.1038/s41467-020-17308-z (PMC7351766; doi:10.1038/s41467-020-17308-z)
Supplement: Supplementary file 1 — Supplementary Information [file 41467_2020_17308_MOESM1_ESM.pdf]

SUPPLEMENTARY INFORMATION

Cryo-EM structure of catalytic ribonucleoprotein complex RNase MRP

Anna Perederina et al.

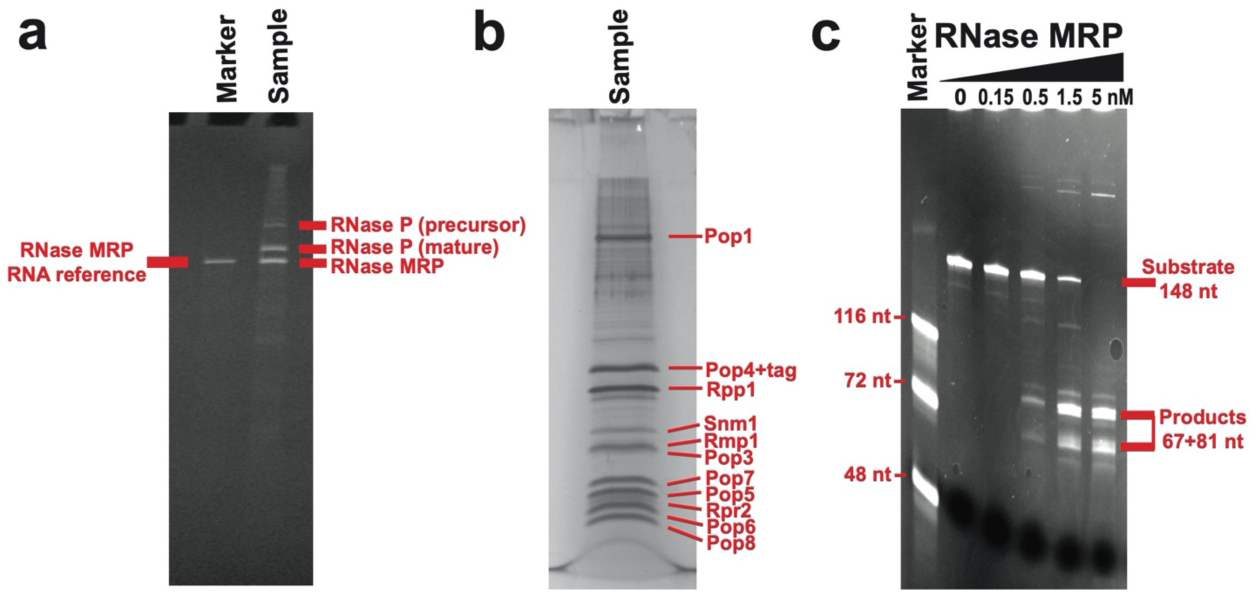

Supplementary Fig. 1 Isolation of RNase MRP.
(**a**) RNA components in the isolated sample. Ethidium bromide stained denaturing PAAG.
(**b**) Protein components as identified by mass spectrometry. Silver stained SDS PAAG.
(**c**) RNase MRP cleavage of a fragment of the internal transcribed spacer 1 (ITS1) of the rRNA precursor containing the A3 site[1]. The substrate (400 nM) was digested by 0; 0.15 nM; 0.5 nM; 1.5 nM; 5.0 nM of RNase MRP (as shown above the gel) for 60 min at 30^0C in a buffer containing 20 mM Tris-HCl (pH 7.5), 50 mM NaCl, 10 mM $MgCl_2$, 1 mM DTT, 50 μg/ml BSA. Ethidium bromide stained denaturing PAAG.

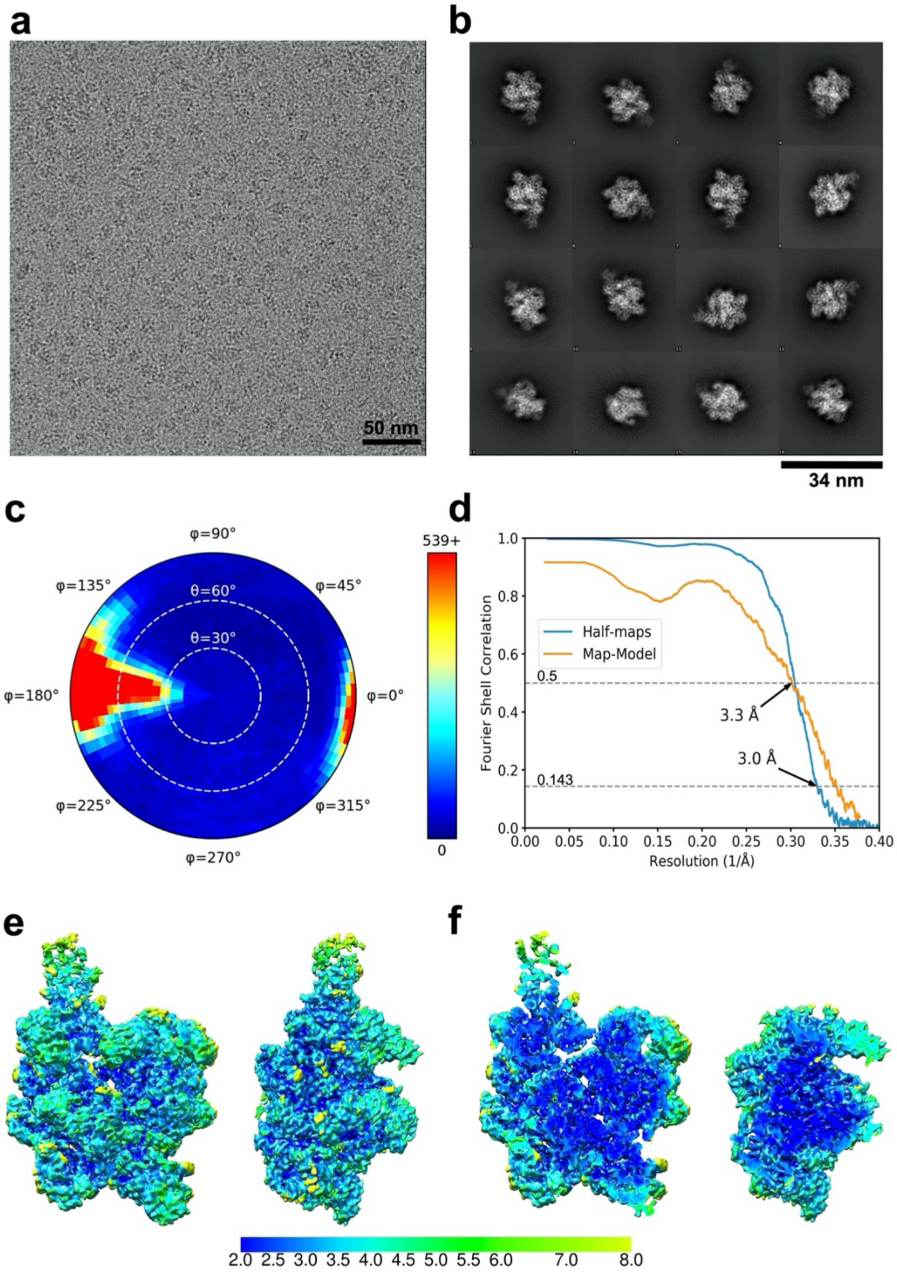

Supplementary Fig. 2 Cryo-EM data collection and processing.
(**a**) A representative cryo-EM micrograph.
(**b**) Representative 2D class averages for RNase MRP particles.
(**c**) The distribution of the Euler angles for the aligned particles used in the final RNase MRP reconstruction. While there was a substantial orientational bias, it did not have a noticeable effect on the quality of the map. Note that a similar or stronger bias was observed for yeast, human, and archaeal RNases P[2,3,4].
(**d**) Fourier shell correlation curves (FCS) for the final map and model. The gold-standard FCS curve is shown in blue; the final model to the final map FSC curve is shown in orange.
(**e, f**) Local resolution map (surface and with the top half removed, correspondingly). The resolution (in Å) is color-coded according to the bar below the map.

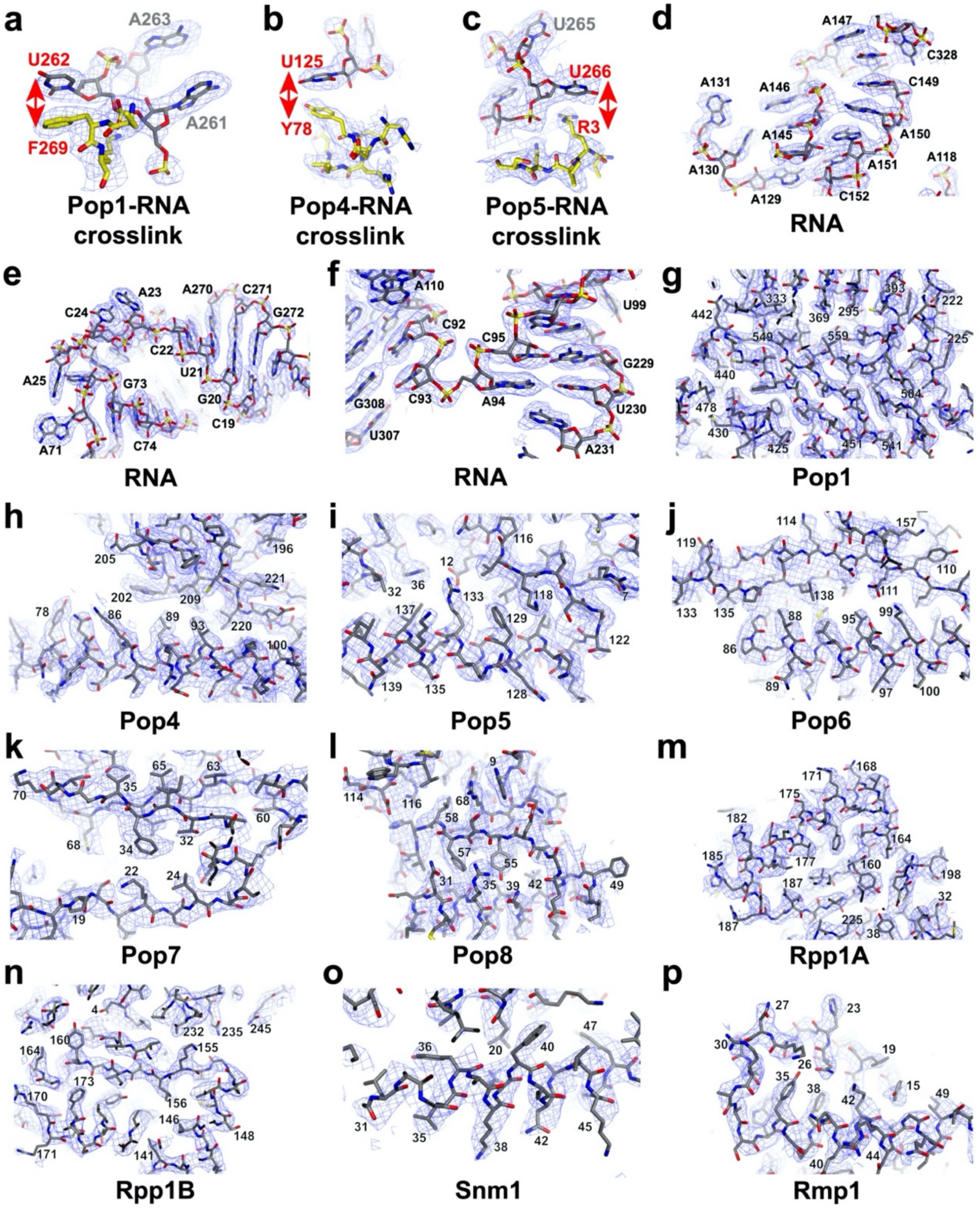

Supplementary Fig. 3 Representative fragments of the final map used in the model building and refinement.

(**a-c**) Fragments corresponding to the previously identified UV-induced RNA-protein crosslinks (shown by red arrows)[5].

(**d-f**) Representative fragments of the RNase MRP RNA component.

(**g-p**) Representative fragments of RNase MRP proteins Pop1, Pop4, Pop5, Pop6, Pop7, Pop8, Rpp1 (copies A and B), Snm1, and Rmp1, as marked below the panels.

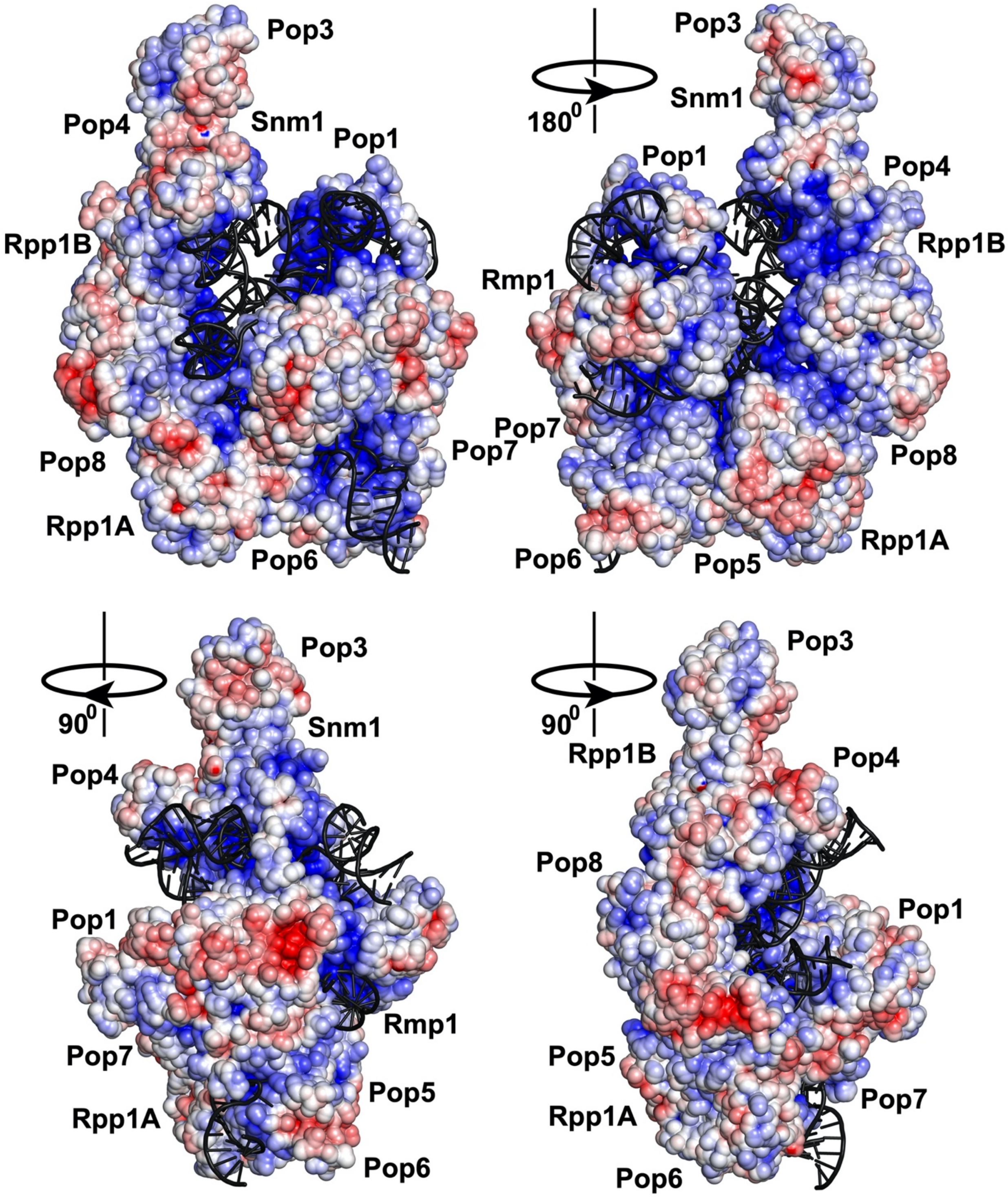

Supplementary Fig. 4 Surface electrostatic potential distribution for the RNase MRP proteins and the RNA component of RNase MRP.

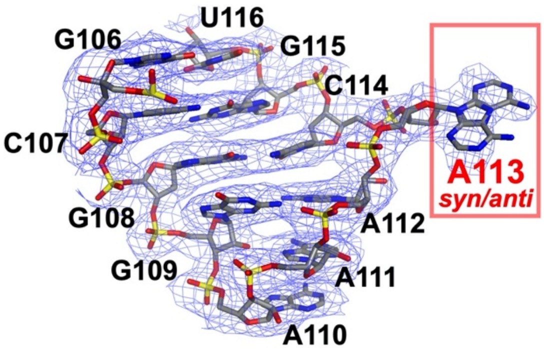

Supplementary Fig. 5 The structure of the phylogenetically conserved RNase MRP RNA element 5'-GA(G/A)A(G/A)-3' (nucleotides 109-113) and the density. Conserved purine A113 that is observed in both syn- and anti- conformations is shown boxed.

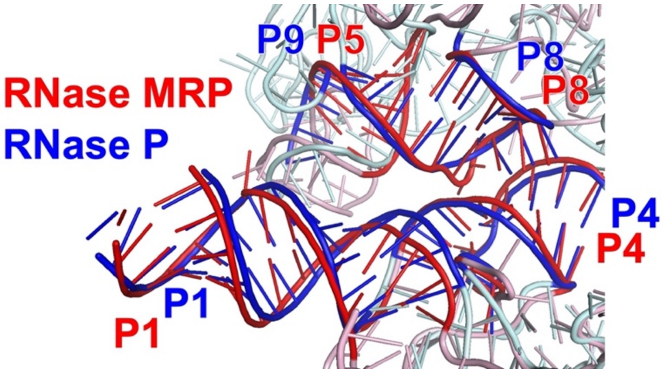

Supplementary Fig. 6 The docking of the P8/P5 helical stack against the P1/P4 stack in RNase MRP is similar to the docking of the P8/P9 stack against the P1/P4 stack in RNase P. RNase MRP stem P5 is found in the position occupied by stem P9 in RNase P, while RNase MRP stem P8 sits in the same position as stem P8 in RNase P. RNase MRP RNA is shown in red; RNase P RNA[2] is shown in blue.

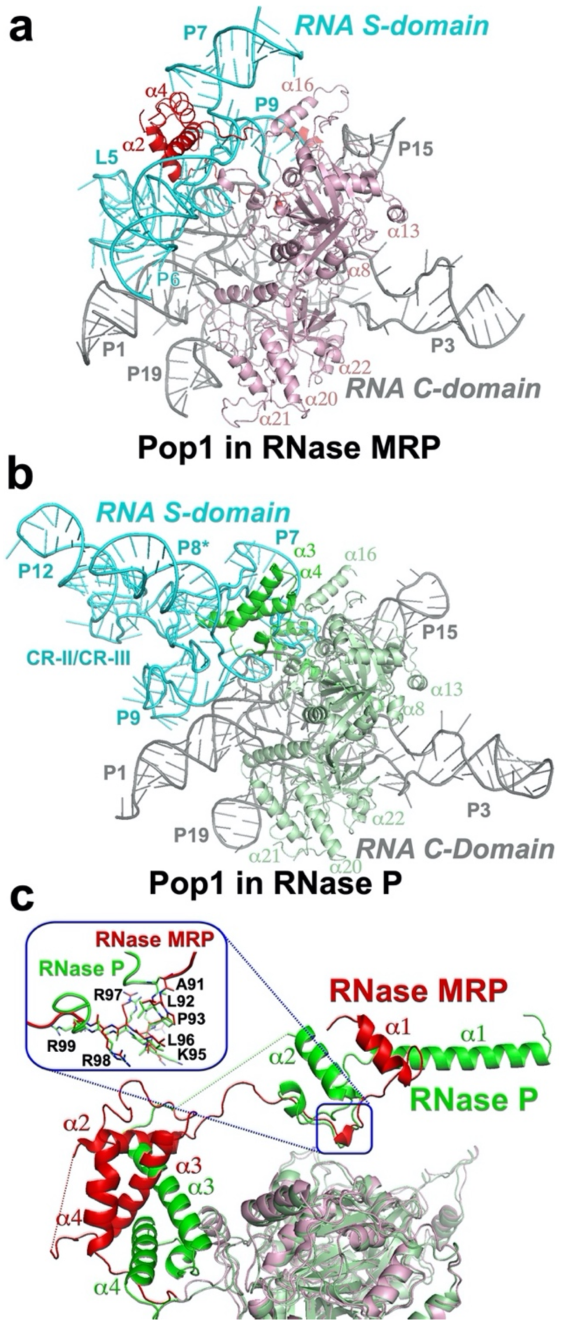

Supplementary Fig. 7 Protein Pop1 in RNase MRP and RNase P.
(**a, b**) The N-terminal part of Pop1 (shown in red for RNase MRP and green for RNase P) interacts predominantly with the S-domains of RNase MRP (panel A, shown in cyan) and RNase P[2] (panel B, shown in cyan), whereas the C-terminal part of Pop1 (residues 203-875, shown in pink and pale green for RNase MRP and RNase P, correspondingly) interacts with the C-domains of RNase MRP and RNase P (shown in grey).
(**c**) Superposition of Pop1 in RNase MRP (red and pink) and Pop1 in RNase P (green and pale green). The folds of the N-terminal parts (red and green for RNase MRP and RNase P, respectively) differ significantly, with the exception of the residues 91-99, where the two folds converge (insert).

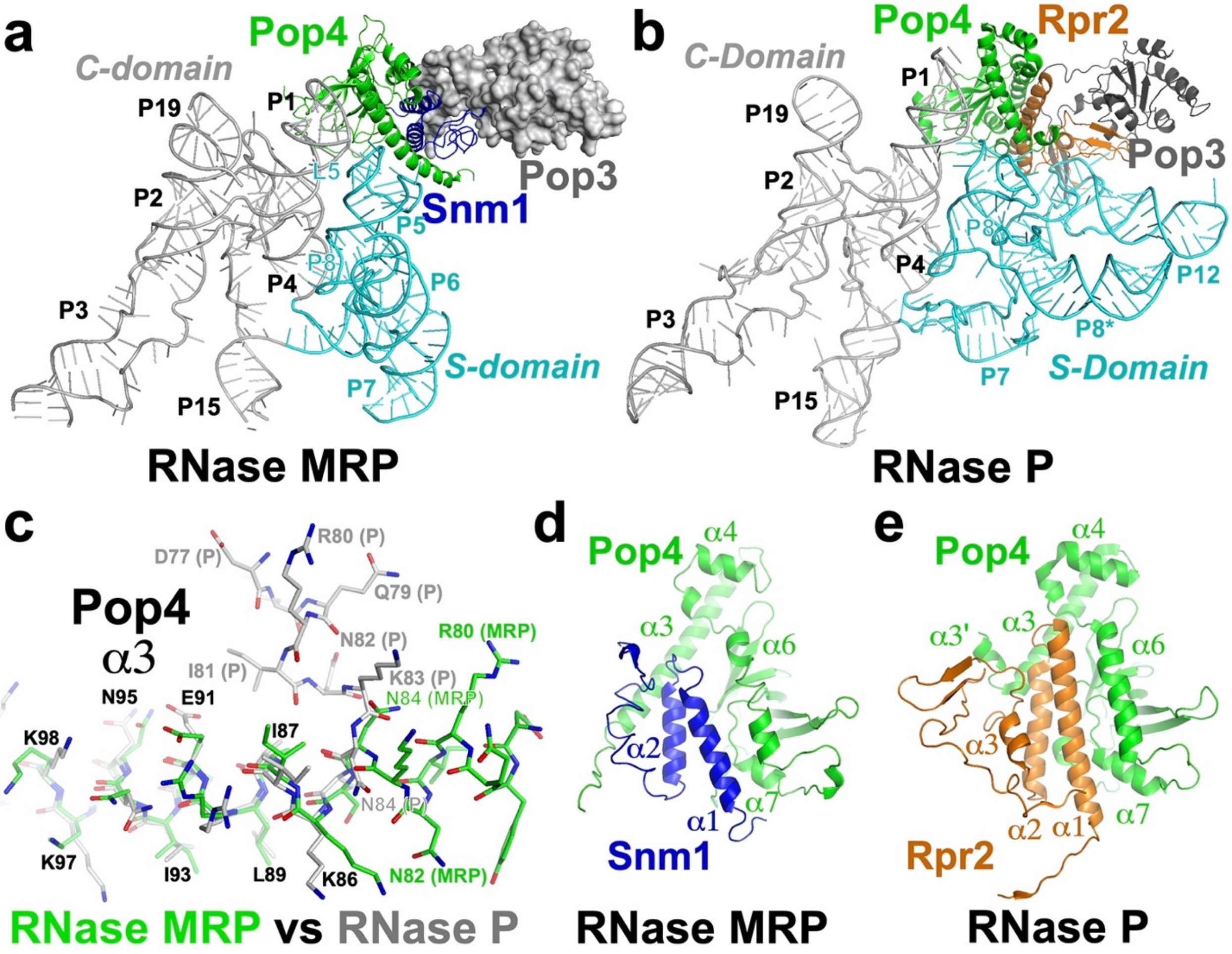

Supplementary Fig. 8 Proteins Pop4, Snm1, Rpr2, Pop3 in RNase MRP and RNase P.
(**a**) In RNase MRP, Pop4 (green) bridges stem P1 of the C-domain RNA (grey) and P5/L5 stem/loop of the S-domain RNA (cyan) and interacts with Snm1 (blue) and Pop3 (grey).
(**b**) In RNase P[2], Pop4 (green) bridges stem P1 of the C-domain RNA (grey) and P9 stem of the S-domain RNA (cyan) and interacts with Rpr2 (orange) and Pop3 (grey).
(**c**) The α3 helix of Pop4 in RNase MRP is disrupted in RNase P. The α3 helix in RNase MRP is shown in green; the corresponding part of Pop4 in RNase P is shown in grey.
(**d, e**) Interactions of RNase MRP protein Snm1 (blue) with Pop4 (green) in RNase MRP (d) versus interactions of RNase P protein Rpr2 (orange) with Pop4 (green) in RNase P (e).

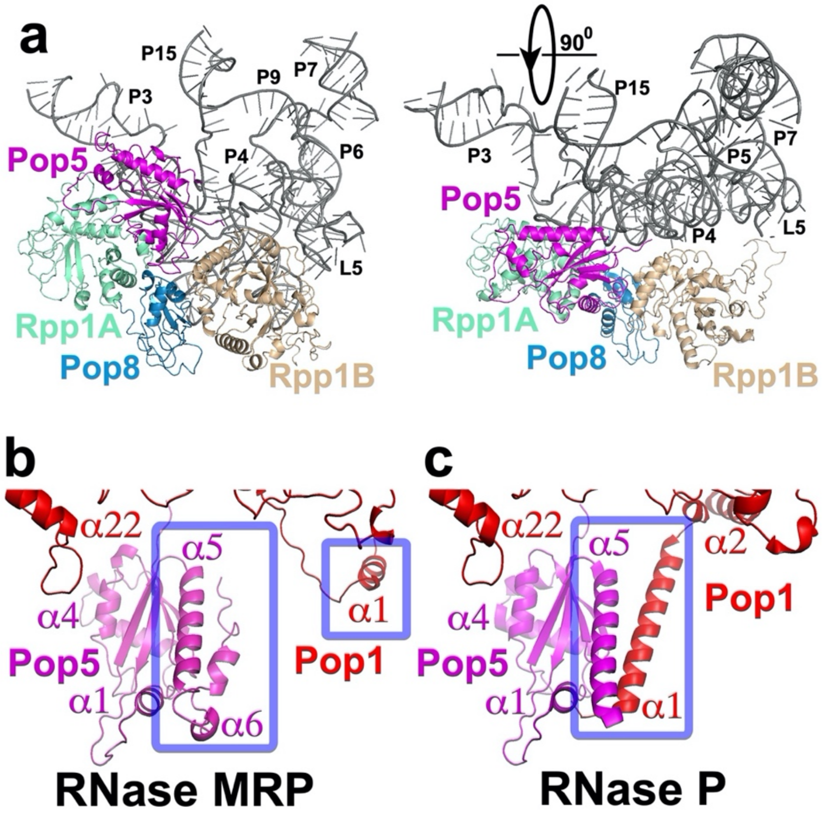

Supplementary Fig. 9 Proteins Pop5, Pop8, Rpp1 in RNase MRP.
(**a**) Interactions of the Pop5/Pop8/$Rpp1_{(A,B)}$ heterotetramer with the RNA component of RNase MRP. Proteins are colored as shown.
(**b, c**) Differences in the Pop5 fold and interactions with Pop1 in RNase MRP (b) versus RNase P[2] (c). The divergent parts are shown boxed; Pop5 is shown in magenta; Pop1 is shown in red.

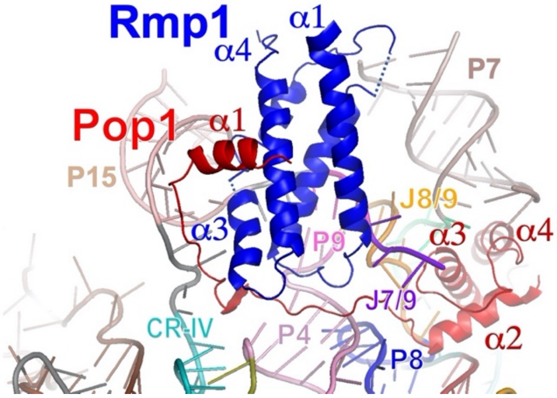

Supplementary Fig. 10 Protein Rmp1 interacts with the N-terminal elements of Pop1 and RNase MRP RNA. Rmp1 is shown in blue; Pop1 is shown in red; RNase MRP RNA is shown in colors matching Fig. 1a.

	RNase MRP (EMD-21564) (PDB 6W6V)
**Data collection**	
EM equipment	FEI Titan Krios
Detector	Falcon 3EC
Pixel size (Å)	0.886
Electron exposure (e^{-}/Å^2)	39
Defocus range (µm)	-1.1 ~ -2.7
**Reconstruction**	
Software	cisTEM-1.0.0
Number of used particles	155,205
Map resolution (Å)	3.0
FSC threshold	0.143
**Model building**	Coot 0.8.9
**Refinement**	
Software	Phenix 1.18.1
**Model composition**	
Chains	11
Non-hydrogen atoms	24,850
Protein residues	2306
RNA nucleotides	294
**Validation**	
R.m.s. deviations	
Bond lengths (Å) (# >4σ)	0.004 (0)
Bond angles (°) (# >4σ)	0.623 (2)
Ramachandran plot	
Favored (%)	89.06
Allowed (%)	10.94
Disallowed (%)	0.0
MolProbity score	1.94
Clashscore	6.61

Supplementary Table 1 Cryo-EM data collection, refinement and validation statistics.

Supplementary references

1. Lygerou, Z., Mitchell, P., Petfalski, E., Seraphin, B. & Tollervey, D. The POP1 gene encodes a protein component common to the RNase MRP and RNase P ribonucleoproteins. *Genes Dev.* **8**, 1423-1433 (1994).

2. Lan, P., Tan, M., Zhang, Y., Niu, S., Chen, J., Shi, S., Qiu, S., Wang, X., Peng, X., Cai, G., Cheng, H., Wu, J., Li, G. & Lei, M. Structural insight into precursor tRNA processing by yeast ribonuclease P. *Science* **362**, pii: eaat6678 (2018).

3. Wu, J., Niu, S., Tan, M., Huang, C., Li, M., Song, Y., Wang, Q., Chen, J., Shi, S., Lan, P. & Lei, M. Cryo-EM Structure of the Human Ribonuclease P Holoenzyme. *Cell* **175**, 1393-1404 (2018).

4. Wan, F., Wang, Q., Tan, J., Tan, M., Chen, J., Shi, S., Lan, P., Wu, J. & Lei, M. Cryo-electron microscopy structure of an archaeal ribonuclease P holoenzyme. *Nat. Commun.* **10**, 2617 (2019).

5. Khanova, E., Esakova, O., Perederina, A., Berezin, I. & Krasilnikov, A.S. Structural organizations of yeast RNase P and RNase MRP holoenzymes as revealed by UV-crosslinking studies of RNA-protein interactions. *RNA* **18**, 720-728 (2012).
